# Preparation of Quinolinium Salts Differing in the Length of the Alkyl Side Chain

**DOI:** 10.3390/molecules17066386

**Published:** 2012-05-25

**Authors:** Jan Marek, Vladimir Buchta, Ondrej Soukup, Petr Stodulka, Jiri Cabal, Kallol K. Ghosh, Kamil Musilek, Kamil Kuca

**Affiliations:** 1 Department of Toxicology, Faculty of Military Health Sciences, University of Defence, Hradec Kralove, 50001, Czech Republic; 2 Department of Epidemiology, Faculty of Military Health Sciences, University of Defence, Hradec Kralove, 50001, Czech Republic; 3 Department of Clinical Microbiology, University Hospital, Hradec Kralove, 50005, Czech Republic; 4 Department of Public Health, Faculty of Military Health Sciences, University of Defence, Hradec Kralove, 50001, Czech Republic; 5 Centre for Biomedical Research, University Hospital, Hradec Kralove, 50005, Czech Republic; 6 Centre of Advanced Study, Faculty of Military Health Sciences, University of Defence, Hradec Kralove, 50001, Czech Republic; 7 School of Studies in Chemistry, Pt. Ravishankar Shukla University Raipur, Raipur, 492010, India; 8 Department of Chemistry, Faculty of Science, University of Hradec Kralove, Rokitanskeho 62, Hradec Kralove, 50003, Czech Republic

**Keywords:** quinolinium salts, synthesis, HPLC, surfactants, detergents, disinfectants, *in vitro* antimicrobial activity

## Abstract

Quaternary quinolinium salts differing in alkyl chain length are members of a widespread group of cationic surfactants. These compounds have numerous applications in various branches of industry and research. In this work, the preparation of quinoline-derived cationic surface active agents differing in the length of the side alkyl chains (from C_8_ to C_20_) is described. An HPLC method was successfully developed for distinction of all members of the series of prepared long-chain quinolinium derivatives. In conclusion, some possibilities of intended tests or usage have been summarized. *In vitro* testing using a microdilution broth method showed good activity of a substance with a C12 chain length against Gram-positive cocci and *Candida* species.

## 1. Introduction

Cationic surfactants usually consist of a hydrophilic part represented by quaternary nitrogen moiety and a hydrophobic part represented by a long alkyl chain. They can be used in various branches of industry and in analytical or physical chemistry. However the use of these agents is very widespread and much more practical applications have been discovered. The most useful analogues are benzalkonium and pyridinium salts [1]. Some of the pyridinium salts have already been used as disinfectants in various technological applications (e.g., components of eye drops, disinfectant solutions, disinfectant foams) and their surfactant characteristics (e.g., critical micellar concentration, surface tension) have already been determined and published [2]. Some of the pyridinium salts (C_12_ and C_16_) were used to solubilize water-insoluble compounds in analytical chemistry applications, where these can also serve as a qualitative and quantitative tool [3]. There are other interesting applications of cationic surfactants such as their addition to chewing gums as an antiplaque agent or other uses in dentistry [4,5]. Pyridinium quaternary compounds are known as competitive inhibitors of acetylcholinesterase [6], analogues with C_10_ and longer chains are also known as nicotinic receptor antagonists [7] and their influence on adsorption of oligonucleotides to a phospholipid membrane was observed [8]. Many non-pyridinium compounds are utilized in the industrial applications such as road construction and repair, enhanced oil recovery and as pigment or corrosion inhibitors [9]. It has been known that these compounds are able to form micelles, which play an important role in the decontamination process [10,11,12]. These formations are created in a water solution, when the critical micellar concentration (CMC) is exceeded. Therefore, many cationic surfactants work as micellar catalysts, *i.e.*, they can accelerate chemical (decomposition) reactions [13,14].

However, this study is focused on an antimicrobial activity of cationic surfactants that were described earlier by several authors [15,16]. Picking one practical application, description of a method using a C_16_ pyridinium as a spray for protecting poultry against bacterial contamination has been reported [17]. Furthermore, a rather potent antimicrobial activity of quinoline analogues (quinolinium stilbene benzenesulfonates) was already described, especially against Gram positive bacteria [18].

The aim of our project was to synthesize quinolinium salts series with different alkyl chains and to test their antimicrobial profile. The compound containing a C_12_ alkyl chain was selected for testing as a representative of the whole series, since benzalkonium compounds with C_12_ side alkyl chain length (together with C_14_ and C_16_) are usually the main components of commercially used disinfectant mixtures. This length of alkyl chain probably allows incorporation of the compound into biological membranes and leads to the lysis of the microbial cell.

## 2. Results and Discussion

### 2.1. Synthesis and HPLC Analysis

Preparation of several quinolinium derivatives has been described before [19]. However, there has been no description for a synthesis of the whole series of such salts differing in alkyl chain length (C_8_ to C_20_). Formerly, a similar method for preparation of benzalkonium and pyridinium salts was reported [20,21]. Therefore, a universal method for preparation of monoquaternary quinolinium salts with chain substituents was developed. Subsequently, a universal HPLC method for analysing quaternary surfactants was developed too.

The results achieved with the prepared compounds are shown in Table 1 (yields, melting points and HPLC retention times). Apparently, the preparation of quinolinium salts with C_8_-C_18_ side chains is relatively trivial, but the yields are rather low compared to the yields of the quaternized *N,N*-dimethyl-*N*-benzylamine or pyridine (previously prepared compounds [20,21]). The larger nucleophilic moiety (quinoline) decreases the probability of a nucleophilic attack. In general, we have obtained a small amount of white crystals of the desired compound from the reaction mistures. Reaction products were detected using the TLC method. Satisfactory purity was reached after one recrystallization from ether. On the other hand, the compound with the C_20_ alkyl group length had to be recrystallized several times to achieve sufficient purity, however the yield decreased rapidly in this case. It was observed that yields decreased with the increased alkyl length.

**Figure 1:**
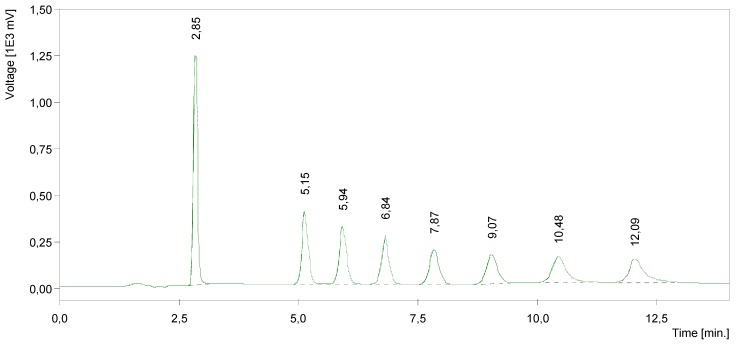
HPLC chromatogram of a quinolinium compound mixture.

Additionally, a HPLC method was developed for product evaluation. It was able to distinguish among all prepared quaternary quinolinium salts (Figure 1). The shortest retention time was found for the C_8_ quinolinium salt. This novel HPLC method could be easily used for characterization of mixtures of such quinolinium compounds.

### 2.2. *In-Vitro* Antimicrobial Activity Assessment 

The antimicrobial efficacy of the synthesized compounds with 12 carbons in the alkyl chain (compound **5**) was chosen and tested using the microdilution broth method. The MICs, MBCs and MFCs were determined for eight fungal and eight bacterial strains. The antibacterial activity is summarized in Table 2. Compound **5** was more effective against Gram-positive bacteria than against Gram-negative bacteria. The effect does not vary substantially in time and a relatively fast effect onset of activity is supposed. MIC values after 48 h were usually equivalent to MBC values.

The antifungal activity is summarized in Table 3. Compound **5** was more effective against *Candida* strains compared to filamentous fungi. The strain *Lichthemia corymbifera* 272 was susceptible only to the highest dose tested (500 μM·L^−1^). The values of MICs for bacterial as well as fungal strains were identical with the MBCs and MFCs suggesting a microbicidal potential of these compounds. This property is desirable for the type of substances such as disinfectants.

## 3. Experimental

### 3.1. General

#### 3.1.1. Synthesis 

**Figure 2:**
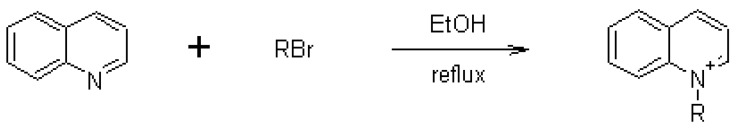
Preparation of quinolinium salts. R = C_8_–C*20* (Table 1).

A universal method for preparation (Figure 2) of monoquaternary quinolinium salts **3**–**9** was developed as follows: pure quinoline (**1**; 1 eq.) in dry ethanol was mixed with the appropriate 1-bromoalkane **2** (14 eq.). The mixture was refluxed for 40 hours. The solution was evaporated under reduced pressure and the crude oily product was recrystallized from ether, filtered, washed with ether and allowed to dry at r.t. The reaction process was followed by TLC (Kieselgel Merck; mobile phase chloroform/methanol 100/1; detection UV 254, Dragendorff reagent).

Yields (%), melting points (Boetius, m.p. were uncorrected) are summarized in Table 1. ^1^H-NMR spectra (Varian Gemini 300, 300 MHz) characterizing each compounds are summarized below:

*N-Octylquinolinium bromide* (**3**). ^1^H-NMR (300 MHz, DMSO) ppm 9.37 (d, *J* = 5.25, 1.37 Hz, 1H), 9.22 (d, *J* = 8.20 Hz, 1H), 8.51 (d, *J* = 7.56 Hz, 1H), 8.30 (d, 1H), 7.95 (d, *J* = 7.62 Hz, 1H), 8.11–8.07 (m, 1H), 8.16 (d, *J* = 8.50 Hz, 1H), 5.09 (t, *J* = 7.55 Hz, 2H), 1.94 (t, *J* = 7.33 Hz, 2H), 1.45–1.08 (m, 10H), 0.80 (t, *J* = 6.59 Hz, 3H).

*N-Decylquinolinium bromide* (**4**). ^1^H-NMR (300 MHz, DMSO) ppm 9.38 (d, *J* = 5.26 Hz, 1H), 9.23 (d, *J* = 8.34 Hz, 1H), 8.51 (d, *J* = 8.18 Hz, 1H), 8.32 (d, *J* = 8.61 Hz, 1H), 8.19 (d, 1H), 8.12–8.07 (m, 1H), 7.95 (d, *J* = 7.62 Hz, 1H), 5.09 (t, *J* = 7.51 Hz, 2H), 1.94 (t, *J* = 7.19 Hz, 2H), 1.44–1.10 (m, 14H), 0.80 (t, *J* = 6.59 Hz, 3H).

*N-Dodecylquinolinium bromide* (**5**). ^1^H-NMR (300 MHz, DMSO) ppm 9.37 (d, *J* = 5.25 Hz, 1H), 9.22 (d, *J* = 8.32 Hz, 1H), 8.51 (d, *J* = 8.21 Hz, 1H), 8.16 (d, *J* = 7.31 Hz, 1H), 7.95 (t, *J* = 7.63 Hz, 1H), 8.12–8.07 (m, 1H), 8.30 (d, 1H), 5.08 (t, *J* = 7.50 Hz, 2H), 1.92 (t, 2H), 1.45–1.09 (m, 18H), 0.81 (t, *J* = 6.48 Hz, 3H).

*N-Tetradecylquinolinium bromide* (**6**). ^1^H-NMR (300 MHz, DMSO) ppm 9.38 (d, *J* = 5.26 Hz, 1H), 9.19 (d, *J* = 8.35 Hz, 1H), 8.51 (d, *J* = 8.16 Hz, 1H), 8.30 (d, *J* = 8.52 Hz, 1H), 8.01 (d, 1H), 8.15–8.09 (m, 1H), 7.94 (d, *J* = 7.61 Hz, 1H), 5.08 (t, *J* = 7.52 Hz, 2H), 1.94 (t, *J* = 7.16 Hz, 2H), 1.47–1.14 (m, 22H), 0.82 (t, *J* = 6.61 Hz, 3H).

*N-Hexadecylquinolinium bromide* (**7**). ^1^H-NMR (300 MHz, DMSO) ppm 9.32 (d, *J* = 5.26 Hz, 1H), 9.18 (d, *J* = 8.38 Hz, 1H), 8.51 (d, *J* = 8.25 Hz, 1H), 8.28 (d, *J* = 3.80 Hz, 1H), 8.05 (d, 1H), 8.12–8.08 (m, 1H), 7.89 (d, 1H), 5.08 (t, *J* = 7.23 Hz, 2H), 1.85 (t, 2H), 1.46–1.11 (m, 26H), 0.82 (t, *J* = 5.94 Hz, 3H).

*N-Octadecylquinolinium bromide* (**8**). ^1^H-NMR (300 MHz, DMSO) ppm 9.33 (d, *J* = 3.96 Hz, 1H), 9.15 (d, *J* = 8.31 Hz, 1H), 8.50 (d, *J* = 8.18 Hz, 1H), 8.20 (d, 1H), 8.09 (d, *J* = 2.70 Hz, 1H), 7.95–8.13 (m, 1H), 8.27 (d, 1H), 5.07 (t, *J* = 7.50 Hz, 2H), 1.93 (t, *J* = 7.05 Hz, 2H), 1.47–1.12 (m, 30H), 0.82 (t, *J* = 6.51 Hz, 3H).

*N-Eicosylquinolinium bromide* (**9**). ^1^H-NMR (300 MHz, DMSO) ppm 9.12 (d, *J* = 3.96 Hz, 1H), 9.31 (d, *J* = 8.38 Hz, 1H), 8.54 (d, 1H), 8.35 (d, *J* = 7.88 Hz, 1H), 8.25 (d, 1H), 8.06–8.14 (m, *J* = 3.03 Hz, 1H), 7.92 (d, *J* = 7.30 Hz, 1H), 5.10 (t, 2H), 2.02 (t, 2H), 1.46–1.21 (m, 34H), 0.83 (t, *J* = 5.94 Hz, 3H).

#### 3.1.2. HPLC Analysis

After preparation of the quinolinium salts, an appropriate HPLC method for their distinction in the mixture was developed. The HPLC system consisted of a P200 gradient pump (Spectra-Physics Analytical, Fremont, CA, USA), a 7125 injection valve – 10 µL loop (Rheodyne, Cotati, WA, USA), a UV1000 detector (Spectra-Physics Analytical), and a CSW Chromatography Station 1.5 software (DataApex, Praha, Czech Republic). A 250 × 4 mm I.D. Waters Spherisorb Cyano (5 μm) column was used (Supelco Inc., Bellefonte, PA, USA) for analysis. The mobile phase consisted of 45% acetonitrile and 55% water. This mixture was prepared as a 0.1 M sodium acetate solution. Finally, the pH was adjusted with acetic acid to 5.0. The samples were delivered isocratically at a flow-rate of 1 mL/min. The absorbance was measured at 257 nm.

### 3.2. *In-Vitro* Antimicrobial Testing

#### 3.2.1. Antifungal Activity

*An in vitro* antifungal activity of the prepared compounds was evaluated on a panel of eight clinical isolates of fungi, four yeasts (*C. albicans* ATCC 44859, *C. krusei* E28, *C. tropicalis* 156, *C. glabrata* 20/I), and four filamentous fungi (*Trichosporon asahii* 1188, *Aspergillus fumigatus* 231, *Lichthemia corymbifera* 272, *Trichophyton mentagrophytes* 445). All strains were part of the collection of fungal strains and were deposited at the Department of Biological and Medical Sciences, Faculty of Pharmacy, Charles University, Hradec Kralove, Czech Republic. The ATCC strains *C. albicans* ATCC 90028, *C. parapsilosis* ATCC 22019, and *C. krusei* ATCC 6258 served as the quality control strains.

All isolates were maintained on Sabouraud dextrose agar prior to being tested. A minimum inhibitory concentration (MIC) was determined by a modified microdilution format of the CLSI M27-A3 and M38-A2 for yeasts and filamentous fungi, respectively [22,23]. Dimethylsulfoxide (Sigma, Prague, Czech Republic) served as a diluent for all compounds and its final concentration did not exceed 2%. RPMI 1640 (KlinLab, Prague, Czech Republic) medium supplemented with l-glutamine and buffered with 0.165 M morpholinepropanesulfonic acid (Sigma-Aldrich, Prague, Czech Republic) to pH 7.0 by 10 M NaOH was used as a test medium. The wells of the microdilution tray contained 200 µL of the RPMI 1640 medium with two fold serial dilutions of the prepared compounds (500–0.49 µmol/L) and were inoculated with 10 µL of suspension. Fungal inoculum in RPMI 1640 was prepared to give a final concentration of 5 × 10^3^ ± 0.2 cfu/mL and 5 × 10^4^ ± 0.5 cfu/mL for yeasts and moulds, respectively. The trays were incubated at 36 ± 1 °C and MIC was read visually and spectrophotometrically (OD 450nm) for filamentous fungi and yeasts respectively after 24 and 48 hours. The MIC values for the dermatophytic strain (*T. mentagrophytes*) were determined after 72 hours and 120 hours. The MICs were defined as 80% inhibition (IC_80_) of the growth of control. A minimum fungicidal concentration (MFC) was established for all compounds tested as a concentration which provided a decrease of a number of colonies by ≥99.9% after subculturing of a 100 µL aliquot of each well with a maximum growth of 20% of control.

#### 3.2.2. Antibacterial Activity

*An in vitro* antibacterial activity of the prepared compounds was tested on a panel of eight bacterial strains (*Staphylococcus aureus* ATCC6538, *S. aureus* MRSA HK5996/08, *S. epidermidis* HK6966/08, *Enterococcus* sp. HK14365/08, *Escherichia coli* ATCC8739, *Klebsiella pneumoniae* HK11750/08, *K. pneumoniae* ESBL HK14368/08, and *Pseudomonas aeruginosa* ATCC9027). The ATCC strains also served as quality control strains, the rest of them were clinical isolates from patients and were deposited at the Department of Biological and Medical Sciences, Faculty of Pharmacy, Charles University, Hradec Kralove, Czech Republic. Before the testing the strains were passaged on Mueller-Hinton Agar (HiMedia, Cadersky-Envitek, Hradec Kralove, Czech Republic).

A minimum inhibitory concentration (MIC) of the prepared compounds was determined by the microdilution broth method modified according to standard M07-A07 [24]. Mueller-Hinton Broth (MH, HiMedia) adjusted to pH 7.4 (±0.2) was used as the test medium. DMSO served as a diluent for all compounds and its final concentration did not exceed 2% in the test medium. The wells of the microdilution tray contained 200 µL of the MH broth with twofold serial dilutions of the compounds (500–0.49 µmol/L) and were inoculated with 10 µL of bacterial suspension. Bacterial inoculum in sterile water was prepared to match 0.5 McFarland scale (1.5 × 10^8^ CFU/mL). The MIC values were read visually after 24 h and 48 h incubation at 36 ± 1 °C. The MIC was defined as a complete inhibition of the growth. A minimum bactericidal concentration (MBC) was established for all compounds tested as a concentration that provided a decrease of a colonies number by ≥99.9% after subculturing of a 100 µL aliquot of each well without a visible growth.

## 4. Conclusions

In conclusion, the developed synthetic protocol seems to be useful for a fast preparation of quinolinium salts. The simplicity of the synthesis becomes even more important with respect to the broad-spectrum and high efficiency antimicrobial activity of the C_12_ representative in our tests. Similar antimicrobial properties are expected for the analogues where the antimicrobial efficacy is supposed (C_14_ and C_16_ analogues). Furthermore, this synthetic procedure seems to be a reliable route of synthesis in semi-industrial laboratories, too. By using such a simple synthetic route, a large number of highly biologically active compounds could be prepared within a short period. Therefore, it could be of a high interest of companies focused on the development on novel detergents. As a larger consequence, a practical HPLC protocol was developed for estimation of quaternary detergent purity.

## Figures and Tables

**Table 1:** Yields, melting points and retention times of prepared quinolinium salts.

Compound	Side alkylating chain (R)	Yield (%)	m.p. (°C)	HPLC Rt (min)
3	C_8_	40.64	66–68	5.15
4	C_10_	34.32	48–50	5.94
5	C_12_	33.71	73–75	6.84
6	C_14_	34.99	98–100	7.87
7	C_16_	22.32	90–92	9.07
8	C_18_	22.16	92–94	10.48
9	C_20_	7,35	98–99	12,09

**Table 2:** *In vitro* antibacterial activity of compound **5**.

Bacterial strains	MIC (μM/L)		MBC (μM/L)
	24 hours	48 hours	48 hours
*Staphylococcus aureus* ATCC6538	15.62	15.62	15.62
*Staphylococcus aureus* MRSA HK5996/08	15.62	15.62	15.62
*Staphylococcus epidermidis* HK6966/08	7.81	15.62	15.62
*Enterococcus sp.* HK14365/08	31.25	62.50	62.50
*Escherichia coli* ATCC8739	125.00	125.00	125.00
*Klebsiella pneumoniae* HK11750/08	125.00	125.00	125.00
*Klebsiella pneumoniae* ESBL HK14368/08	125.00	125.00	125.00
*Pseudomonas aeruginosa* ATCC9027	250.00	250.00	250.00

**Table 3:** *In vitro* antifungal activity of compound **5**.

Fungal strains	MIC (μM/L)		MFC (μM/L)
	24 hours	48 hours	48 hours
*Candida albicans* ATCC 44859	15.62	15.62	15.62
*Candida krusei* E28	15.62	15.62	15.62
*Candida tropicalis* 156	15.62	15.62	15.62
*Candida glabrata* 20/I	7.81	15.62	15.62
*Trichosporon beigelii* 1188	15.62	62.50	62.50
*Aspergillus fumigatus* 231	62.50	125.00	250.00
*Aspergillus corymbifera* 272	500.00	500.00	500.00
*Trichophyton mentagrophytes* 445	31.25	31.25	62.50
